# How can EPR spectroscopy help to unravel molecular mechanisms of flavin-dependent photoreceptors?

**DOI:** 10.3389/fmolb.2015.00049

**Published:** 2015-09-01

**Authors:** Daniel Nohr, Ryan Rodriguez, Stefan Weber, Erik Schleicher

**Affiliations:** Department of Physical Chemistry, Institut für Physikalische Chemie, Albert-Ludwigs-Universität FreiburgFreiburg, Germany

**Keywords:** photoreceptor, EPR spectroscopy, distance determination, radicals, flavoproteins

## Abstract

Electron paramagnetic resonance (EPR) spectroscopy is a well-established spectroscopic method for the examination of paramagnetic molecules. Proteins can contain paramagnetic moieties in form of stable cofactors, transiently formed intermediates, or spin labels artificially introduced to cysteine sites. The focus of this review is to evaluate potential scopes of application of EPR to the emerging field of optogenetics. The main objective for EPR spectroscopy in this context is to unravel the complex mechanisms of light-active proteins, from their primary photoreaction to downstream signal transduction. An overview of recent results from the family of flavin-containing, blue-light dependent photoreceptors is given. In detail, mechanistic similarities and differences are condensed from the three classes of flavoproteins, the cryptochromes, LOV (Light-oxygen-voltage), and BLUF (blue-light using FAD) domains. Additionally, a concept that includes spin-labeled proteins and examination using modern pulsed EPR is introduced, which allows for a precise mapping of light-induced conformational changes.

## Introduction

Electron paramagnetic resonance (EPR) spectroscopy is a well-established spectroscopic method for the examination of the global as well as the local structure of paramagnetic molecules. Although only a minority of proteins is intrinsically paramagnetic, numerous proteins contain paramagnetic molecules in form of stable cofactors (e.g., organic molecules, transition-metal ions, or transition-metal clusters) (Jeschke, 2005). In addition, paramagnetic states can be generated transiently during the course of a reaction of a protein. In such a case, cofactor, amino acid and/or substrate radicals can be generated and thus characterized (Frey et al., 2006). Last, artificial paramagnetic labels (spin labels) can be attached to a specific site of the protein of interest. Among others, small and metastable nitroxide spin labels are commonly used, which can be covalently bound to cysteine residues (Klare and Steinhoff, 2009; Jeschke, 2012). As a consequence, cysteines (and afterwards radicals) can be introduced to virtually any position of interest (commonly named as site-directed spin labeling, SDSL) with the help of established molecular-biology techniques (Berliner et al., 2000; Hubbell et al., 2000; Fanucci and Cafiso, 2006).

EPR spectroscopy is not only helpful to characterize stable paramagnetic states, but is extremely powerful to analyze radical intermediates, which often occur in electron-transfer reactions. Here, EPR methods have one advantage as compared to other methods such as NMR: protein-size restrictions do not apply to EPR because the detection of EPR is limited to the paramagnetic molecule itself and only those parts of a biomolecule that directly interact with it. As a non-invasive or minimally invasive method, EPR allows for the investigation of such systems in a functional state or even under in-cell conditions (Berliner, 2010; Hänsel et al., 2014). During the last decade, electron-electron double resonance (DEER or PELDOR) spectroscopy, a modern pulsed EPR method, became increasingly popular (Jeschke, 2002, 2012; Schiemann and Prisner, 2007; Reginsson and Schiemann, 2011; Hubbell et al., 2013). Here, the strength of the dipolar coupling between two radicals is determined, and from that, the distance between two radicals may be obtained with high accuracy. When combining this method with the aforementioned SDSL, distances (and even orientations) between any position in a protein may be obtained, which makes ELDOR spectroscopy similarly powerful as established methods, such as Förster resonance energy transfer (FRET) spectroscopy.

The focus of this review is to evaluate potential scopes of application of EPR to the emerging field of optogenetics—the genetic encoding of light-sensitive proteins that activate signaling pathways in response to light. The main objective for molecular spectroscopy in this context is to unravel the complex mechanisms of light-active proteins (photoreceptors), from their primary photoreaction to downstream signal transduction.

In principle, the mechanism of photoreceptors can be separated into three parts: (i) the response of the chromophore immediately after light excitation, (ii) signal propagation from the chromophore to the signaling domain (or very crudely expressed: from the center of the protein to its surface), and (iii) activation of the signaling domain. The time scales of these processes range from picoseconds for the first process down to (milli)seconds for the subsequent ones; therefore, various spectroscopic techniques are necessary to disentangle the entire mechanism. Only if all these facts are at hand, rational decisions can be made, which photoreceptor to choose for an optogenetic study, and more importantly, how the respective light-responsive proteins may be genetically encoded. Up to now, in most cases, small libraries of fusion variants with several tens of members were prepared and screened manually for optimized response (Möglich and Moffat, 2010). Additionally, many of these constructs show only partially activation/deactivation upon illumination, and thus, could be further optimized.

Although various other photoreceptors, in particular rhodopsins have been investigated using EPR spectroscopy (Van Eps et al., 2015), and have been successfully implemented as optogenetic transponders (Zhang et al., 2011), this review is focused to provide an overview of recent results from EPR spectroscopy on flavin-containing photoreceptors. The three up to now characterized flavin-based receptor classes can in principle be further divided into the large (~60 kDa) cryptochrome/photolyase (CRY/PL) class, and the small (~20 kDa) modular LOV (Light-oxygen-voltage) and BLUF (blue-light using FAD) domains. From the very beginning, flavin-containing photoreceptors have been identified as important tools for optogenetics, and several applications have been published to date (e.g., Wu et al., 2009; Kennedy et al., 2010; Möglich and Moffat, 2010; Christie et al., 2012). One should keep in mind that since their discovery, molecular spectroscopy of all flavors has been applied to these proteins (a number of recent reviews excellently summarize the results, e.g., Chaves et al., 2011; Zoltowski and Gardner, 2011; Losi and Gärtner, 2012), but only bringing the results of the application of various techniques together resulted in the wealth of knowledge on their (photo)chemistry that we have accumulated up to now.

## Introduction to selected EPR techniques

It is beyond the aim of this review to comprehensively introduce the theory and all practical aspects of EPR spectroscopy; for this purpose, the reader is referred to a number of excellent and in-depth reviews and books (e.g., Weil et al., 1994; Schweiger and Jeschke, 2001; Jeschke, 2005). However, we would like to present a few special EPR techniques that have been proven very helpful when working with light-active flavoproteins. Specifically, continuous-wave EPR (cwEPR) is commonly applied for a first more rough characterization of a stable paramagnetic species; for a more detailed characterization of the local structure of a radical, advanced methods, such as pulsed electron-nuclear double resonance (ENDOR) spectroscopy, are preferably applied. For the characterization of meta-stable paramagnetic intermediates, transient EPR (trEPR) with its high time resolution is for sure the method of choice.

### Steady-state EPR spectroscopy

CwEPR spectroscopy is typically used to determine the **g**-tensor and large anisotropic hyperfine couplings (hfcs) of a stable paramagnetic species. In addition to the in general fast and technically simple measurement, one further advantage of cwEPR originates from the so-called lock-in detection. Here, a modulation of the magnetic-field strength with high frequency is applied, and a subsequent detection of the EPR signal at the same modulation frequency results in a significantly increased signal-to-noise ratio. The downside of this technique is its limitation in time resolution, which is restricted to time scale of several tens or hundreds of microseconds when the most common modulation frequency of 100 kHz is applied.

**Figure 1:**
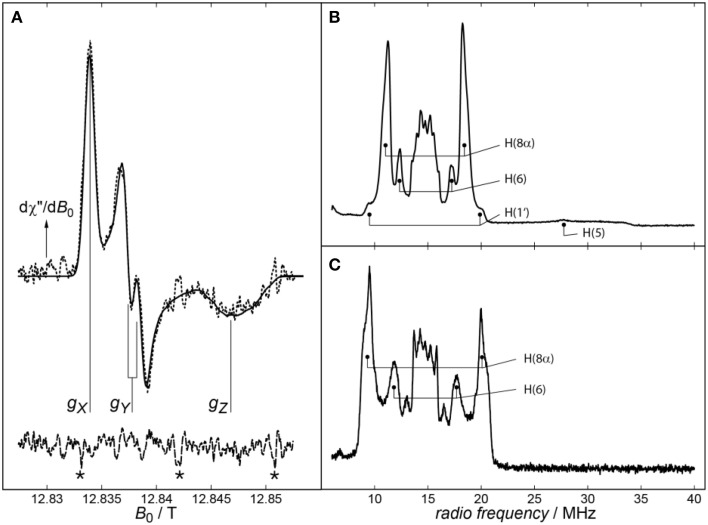
**(A)** cwEPR spectrum of *Xenopus laevis* (6-4) photolyase recorded at 360 GHz. The experimental spectrum and its spectral simulation are depicted as dashed and solid lines, respectively. In addition to the well resolved principal values of the **g**-tensor, a splitting on the *g*_*Y*_ component of the spectrum can be observed, which is due to the projection of the H(5) hfc component *A*_*y*_ onto the *Y* principal axis of **g**. Signals arising from magnetic-field calibration are labeled by asterisks (^*^). Adapted from Schnegg et al. (2006). Right panel: Pulsed proton X-band (Davies) ENDOR spectra from *Synechocystis* sp. **(B)** and *Drosophila melanogaster* **(C)** cryptochromes recorded at 80 K. Assigned proton hfcs are marked. Adapted from Schleicher et al. (2010).

While the isotropic *g*-value of an organic radical can be easily measured at X-band microwave (mw) frequencies, the full resolutions of the **g**-tensor's anisotropy and hyperfine coupling (hfc) parameters typically require high mw frequencies, at least the commercially available Q− and W−band frequencies. Figure 1 (left panel) shows a very high-magnetic-field cwEPR spectrum of *Xenopus laevis* (6-4) photolyase with its protonated FADH^•^ cofactor (dashed line). A least-squares best fit (drawn line) of the experimental data revealed the **g**-tensor principal values and some components of the anisotropic hfcs of N(5), N(10), and H(5). The spectrum was recorded with a laboratory-built EPR spectrometer operating at 360 GHz (Schnegg et al., 2006) because with lower (commercially available) mw frequencies it is impossible to fully resolve the **g**-tensor principal components of flavin radicals (Schleicher and Weber, 2012). Due to the very high magnetic field of 12.8 T, not only the **g**-tensor is fully resolved, but even a splitting of the *g*_y_-component of the **g**-tensor is observed. It originates from the projection of the H(5) hfc component *A*_*y*_ onto the *Y* principal axis of **g**.

**Scheme 1:**
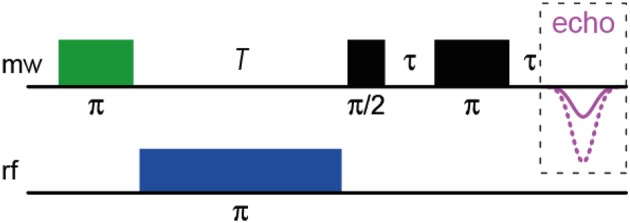
**The Davies-ENDOR pulse sequence**.

Pulsed (Davies-type) ENDOR spectroscopy (for in-depth reviews, see e.g., Prisner et al., 2001; Murphy and Farley, 2006) reveals hfcs of a sample by inducing NMR transitions within a paramagnetic species and surrounding hyperfine-coupled nuclei and detecting them via EPR. For this purpose, the EPR spectrometer is equipped with a radio-frequency (rf) source and amplifier. In comparison to cwEPR, a static magnetic field is applied; its optimal value has to be determined in advance. The Davies-ENDOR pulse sequence (see Scheme 1) then starts with a 180° (i.e., π) mw pulse to invert the magnetization of the electron-spin system, thus generating an “inverted” spin system, in which NMR transitions can be induced by application of a 180° (i.e., π) rf pulse of varying frequency following the mw pulse. In case of resonance, the rf pulse will again invert the magnetization, thus reducing the net magnetization. The pulse sequence is finished by a standard Hahn echo (π/2 – π) sequence. The final spectrum shows the inverted echo intensity as function of the radio frequency. This allows a direct readout of the type of coupling nucleus by its nuclear Larmor frequency. Some limitation of this technique is due to the relaxation time of the electron-spin system, which has to be long enough to apply the rf pulse that is relatively long as compared to the mw pulses; however, this is only a minor problem when working with organic radicals.

Importantly, hfcs are, via the electron-spin density, quite sensitive to changes in their environment, and thus, altered hfcs can be used to gain structural information on the close surroundings of a paramagnetic molecule. In general, hfcs of a particular nucleus can be directly read out from ENDOR spectra as pairs of resonance lines that are, according to the condition ν_ENDOR_ = |ν_N_ ± *A*/2|, either equally spaced about the magnetic-field dependent nuclear Larmor frequency ν_N_ and separated by the hfc constant *A* (for the case ν_N_ > |*A*/2|), or centered around *A*/2 and separated by 2ν_N_ (for ν_N_ < |*A*/2|). A typical proton ENDOR spectrum of a flavin radical at X-band microwave frequency can be divided into five parts: (1) the strong central matrix-ENDOR signal reaching from around 13–16 MHz, originating from weakly coupled protons like backbone or water protons close to the flavin, or those directly attached to the isoalloxazine ring, namely H(3), H(7α) and H(9). (2) Flanking the matrix-ENDOR signal couplings at around 12 and 17 MHz that are assigned to the proton H(6) can be observed. (3) The most intensive features in a flavin radical ENDOR spectrum arise from the protons of the methyl group at C(8), which can be detected at 10–12 MHz and 17–19 MHz. (4) In most published spectra, one of the two β-protons attached to C(1′) of the ribityl side chain is visible as small shoulders at 9–10 and 19–20 MHz (Schleicher et al., 2010). (5) In the neutral protonated state of the flavin radical, a broad rhombic feature can be observed reaching from 20 up to 34 MHz, which is assigned to the proton attached to N(5).

Two exemplary proton ENDOR spectra, one originating from the FADH^•^ of *Synechocystis* sp. CRY DASH, and the other from the FAD^•−^ of *Drosophila melanogaster* CRY, are shown in the right panel of Figure 1. As a first result, the protonation state of the flavin radical can be directly read out of the respective ENDOR spectrum: Depending on the presence or absence of a broad signal between 20 and 34 MHz originating from H(5), deprotonated anionic or protonated neutral flavin radical states, respectively, can be easily distinguished. Additionally, significantly different proton hfcs [in particular H(8α) and H(6)] can be observed upon transforming the anionic FAD^•−^ into the neutral FADH^•^ radical. This is because protonation at N(5) results in a significant redistribution of the unpaired electron-spin density from the less polar xylene ring of the isoalloxazine moiety toward the pyrazine and pyrimidine rings. In sum, proton ENDOR spectroscopy allows for an easy discrimination between the two protonation states, and gives access to the molecular wave function of a paramagnetic molecule via the determination of electron-spin densities (that are directly related to the respective hfcs).

### Transient EPR spectroscopy

Short-lived paramagnetic intermediates, such as triplet states or radical pairs (RP) generated in the course of photo-chemical reactions, can be favorably studied by measuring the EPR signal intensity as a function of time at a fixed magnetic field (Stehlik and Möbius, 1997; Bittl and Weber, 2005; van der Est, 2009). Typically, the best-possible time response of a commercial EPR spectrometer is in the order of about 20 μs (see chapter “Steady-State EPR Spectroscopy”). By removing the magnetic-field modulation, the time resolution can be pushed into the 10^−8^–10^−9^ s range presupposed a suitably fast data acquisition system is present to directly record the transient EPR signal amplitude as a function of time. In transient EPR spectroscopy (trEPR), paramagnetic species are generated by a nanosecond laser flash, which also serves as a trigger signal. Spectral information can be obtained from a series of trEPR signals recorded at various magnetic-field points, thus yielding a two-dimensional variation of the signal intensity with respect to both the magnetic field and the time. Similar to e.g., transient absorption spectroscopy, trEPR spectra can be extracted at any fixed time after the laser pulse as slices parallel to the magnetic-field axis.

**Figure 2:**
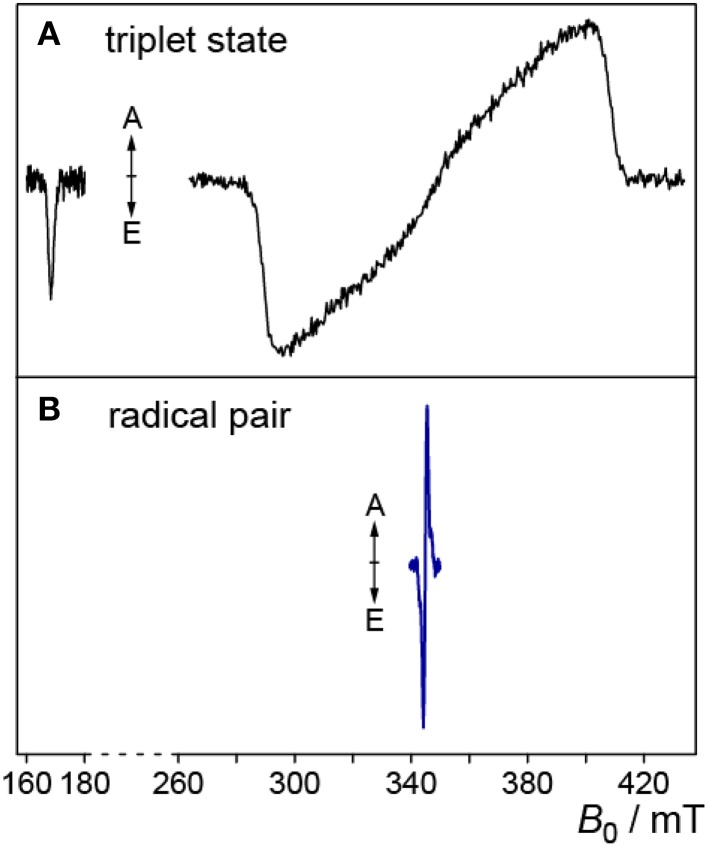
**Triplet and RP trEPR spectra of flavoproteins**. **(A)** Low-temperature (80 K) trEPR spectrum of the photoexcited triplet state of FMN extracted from a full 2D-plot, for details see Kowalczyk et al. (2004). The weak transition at ~170 mT originates from the “Δ*M*_*S*_ = ±2”-transition. **(B)** trEPR spectrum of a photogenerated RP comprising a flavin and a tryptophan radical, measured at 274 K (Biskup et al., 2009).

In Figure 2A, the one-dimensional representation of the trEPR signal from the photo-generated triplet state of FMN is shown (Kowalczyk et al., 2004; Schleicher et al., 2004). Due to signal detection in the absence of any source modulation, the sign of the resonances directly reflects the emissive (E) or enhanced absorptive (A) spin polarization of the EPR transitions, which arises due to the generation of the electron-spin state with an initial non-equilibrium energy-level population (Turro et al., 2000; Woodward, 2002; Hirota and Yamauchi, 2003). The spectral width of the signal reflects the strong mutual interaction of the unpaired electron spins in the triplet configuration. Because they are both localized on the same molecule (in this example on the isoalloxazine moiety of FMN), the spin–spin interactions are strong and hence, trEPR spectra of flavin triplet states are rather broad (Kowalczyk et al., 2004). The weak transition at low magnetic fields arises from the only weakly allowed “Δ*M*_*S*_ = ±2”-transition.

In RPs, the average distance between the two unpaired electron spins is typically much larger. Hence, trEPR-spectra of photo-generated (and electron-spin polarized) RP states are narrower due to the reduced mutual dipolar and exchange interactions as compared to flavin triplet states (Schleicher et al., 2009). In Figure 2B, the trEPR signal of a flavin–tryptophan-based RP with a distance of ~20 Å is depicted. The drastic influence of the strength of electron-electron interactions on the width of the spectra is obvious.

Analysis of the spectral shapes of trEPR-signals yields information on the chemical nature of the individual radicals composing the RP, and their interaction with each other and with their immediate surroundings. Spectral simulations based on the correlated-coupled RP mechanism are typically performed; these are outlined in more detail elsewhere (Closs et al., 1987; Hore, 1989; Kothe et al., 1991; van der Est, 2009). Briefly, the signal of a single pair of coupled radicals consists of four resonance lines arranged in two antiphase doublets, each centered at the resonance magnetic-field position of the individual radical, respectively. The spacing between the lines within the antiphase signals is determined by the exchange and/or dipolar interactions. Each line pair may be further split by hyperfine interactions. In non-oriented frozen samples, interaction anisotropies will contribute to an inhomogeneous spectral broadening of some or all transitions.

## State of the art: intermediates in flavin-dependent photoreceptors probed by modern EPR spectroscopy

### Cryptochromes

CRYs are blue-light photoreceptors with a wide range of regulatory functions in plants, animals and microorganisms. They are closely related to photolyases (PLs), with tight homologies in amino-acid sequence and spatial structure (Essen, 2006; Müller and Carell, 2009). PLs catalyze the light-driven, enzymatic cleavage of certain UV-induced lesions in DNA (Sancar, 2004; Weber, 2005; Essen and Klar, 2006). Except in mammals, PLs are very common in all three kingdoms of life (Essen and Klar, 2006). Both proteins use a flavin adenosin dinucleotide (FAD) as their primary redox-active chromophore/cofactor that is non-covalently bound in a cavity of the protein (Möglich et al., 2010; Chaves et al., 2011; Losi and Gärtner, 2012). Some of the characterized proteins carry an additional chromophore for light harvesting. In PLs, these second chromophores range from methenyltetrahydrofolate (Essen and Klar, 2006; Klar et al., 2006; Müller and Carell, 2009) via flavin derivatives to recently identified ribolumazine (Geisselbrecht et al., 2012; Zhang et al., 2013); however, the relevance for CRY photochemistry and function is still under debate (Selby and Sancar, 2012).

CRYs can function as classical photoreceptors or work as light-independent transcription factors (Lin and Todo, 2005). Examples are flower development and the entrainment of the circadian rhythm (Guo et al., 1998; van der Horst et al., 1999; Chaves et al., 2011). CRYs have also been suggested to play a major role in the magnetic orientation system of migratory birds, fruit flies and other animals (Gegear et al., 2008; Hore, 2012). Both classes of proteins show similarities in their basic structure, which are divided into an N-terminal domain and a C-terminal chromophore-binding domain where the FAD binding site is located. Additionally, CRYs contain an extended C-terminal region, the so-called C-terminal tail (CTT). Its length is rather variable; usually it is longer in plant CRYs than in animal CRYs, and it is supposedly involved in signal transduction. In the recently solved full-length structure of fruit fly CRY, the CTT forms an α-helix which occupies the FAD access cavity, at which the DNA lesion binds in PLs (Czarna et al., 2013).

After light excitation, PLs are able to engage in two light-dependent reactions: If the FAD cofactor is in its fully reduced FADH^−^ state, an electron can be transferred to the DNA lesion, which results in splitting of the damaged DNA. Subsequent electron transfer back to the FAD renders this reaction net-zero with respect to the number of exchanged electrons. On the other hand, PLs and CRYs are able to react in the so-called photoreduction reaction: If the FAD is not in its fully reduced FADH^−^ state, electrons can be transferred from the surface of the protein to the FAD by receiving an electron from a proximal tryptophan. Trp306 (*E. coli* PL numbering), which is located at the surface of the protein and is about 20 Å apart from the FAD, was first identified via a point-mutational study (Li et al., 1991). This distance is too large for an efficient direct electron transfer, and soon thereafter, two additional tryptophans (Trp359 and Trp382) were discovered as part of the so called “tryptophan triad” (Trp-triad) (Li et al., 1991). This triad represents a highly conserved electron-transport chain, which can be found in almost all members of the CRY/PL family. After the first electron transfer step, the electron hole is transported via well-defined intermediates from the proximal tryptophan to the surface-exposed (terminal) tryptophan, which in turn results in a transiently formed RP between the FAD and the terminal tryptophan. This intermediate state could either recombine back to the ground state, or is further stabilized via a subsequent deprotonation of the tryptophanyl cation radical involving solvent water molecules to form a neutral tryptophanyl radical (Aubert et al., 2000). The life time of the secondary RP comprising of FAD^•−^ (or FADH^•−^ in case of plant CRYs and PLs) and Trp^•^ is significantly prolonged up to the millisecond range. If the neutral tryptophanyl radical is reduced by an external electron donor, then the FAD remains in a one-electron reduced state. By substitution mutations it could be shown that both the surface-exposed tryptophan as well as the two bridging tryptophans are crucial for photoreduction ability. In PLs, however, the relevance of this intraprotein reaction for physiological DNA repair has been questioned (Kavakli and Sancar, 2004).

Despite the CTT region, CRY and PL structures are highly homologous, and although both harbor FAD as their redox-active chromophore, it seems that CRYs lost their ability to repair DNA damages. Earlier studies pointed out that the resting state *in vivo* in PLs is the fully reduced FADH^−^ (Payne et al., 1987). To elucidate if the *in vivo* redox state is different in CRYs, insect cells overexpressing various CRYs were measured in the dark and after blue-light illumination using UV-vis and EPR spectroscopy. Oxidized FAD can in principle be detected in cells by fluorescence spectroscopy (by monitoring the emission at 525 nm, Galland and Tölle, 2003) or by UV-vis spectroscopy (by monitoring the absorbance at ~450 nm). It could be shown that a light induced absorbance change matching the reduction of oxidized FAD occurs after a few minutes of blue-light illumination. The drawbacks of these optical methods are that (i) direct concentration measurements of the illuminated FAD states are not possible, and (ii) large experimental uncertainties arise from the intrinsic absorbance (and/or fluorescence) of cells. On the other hand, the use of EPR spectroscopy enabled direct measurements of the formation of the semiquinone radicals in living cells. Specifically, dark-grown intact cells overexpressing various CRYs were illuminated for defined time intervals with blue light, and were subsequently shock-frozen in liquid nitrogen (Banerjee et al., 2007; Bouly et al., 2007; Hoang et al., 2008). The increase of an organic radical signal could be detected after a few minutes of illumination; control cells did not show any radical signal. Furthermore, a decrease of the signal back to a complete decay could be measured after dark incubation of the samples overnight (Engelhard et al., 2014).

To further characterize the detected radical signatures, and to assign the signal to a specific molecule, (Davis) ENDOR was applied at 80 K. Clearly, resonances could be obtained that are highly similar to those of purified protein-bound FAD^•−^ and FADH^•^ radicals found in plant and animal cryptochromes, respectively. With these results at hand, a novel mechanism of plant and animal CRYs could be proposed: In contrast to PLs, CRYs are activated via photoreduction of FAD starting from the oxidized FAD state (Banerjee et al., 2007; Bouly et al., 2007; Hoang et al., 2008). The formation of the fully-reduced FADH^−^ state is strongly inhibited.

As a next logical step, the intermediate RPs that appear during the photoreduction reaction have been investigated in more detail. To this end, a trEPR study of wild-type CRY DASH of *X. laevis* (*X*Cry) was conducted to shed light on light-induced paramagnetic intermediates (Biskup et al., 2009). The obtained spectrum of wild-type *X*Cry resembles those published previously from trEPR on light-induced short-lived RP species in FAD photoreduction of PLs (Weber et al., 2002; Weber, 2005). The origin of the RP signature in *X*Cry could be unraveled by examination of a point mutant, Trp324Phe, which lacks the terminal tryptophan (equivalent to Trp306 in *E. coli* CPD photolyase) of the conserved electron-transfer cascade. This mutant did not show any trEPR signal. The conclusion, that Trp324 is indeed the terminal electron donor in electron-transfer reaction in *X*Cry was further supported by spectral simulations, which have been performed on the basis of the correlated coupled RP model. The calculations were performed using published **g**-tensor parameters for FAD and tryptophan neutral radicals. The relative orientations of the principal axes of both **g**-tensors and the dipole–dipole coupling tensor were taken from a *X*Cry homology model and kept fixed (Biskup et al., 2009).

After assigning the trEPR signal of the WT to the RP [FAD·· Trp324], the role of the first two tryptophans of the triad, namely Trp377 and Trp400 was explored in more detail (Biskup et al., 2013). For this purpose, two additional mutants, Trp377Phe and Trp400Phe, were produced and examined under otherwise identical experimental conditions. In contrast to the Trp324Phe sample, trEPR signals could be—most unexpectedly—observed in both mutants. At first glance, the obtained trEPR signal patterns are similar to the ones from the WT; however, slight differences could be detected upon closer inspection: Most importantly, the signals are shifted toward lower magnetic field, which corresponds to at least one paramagnetic species with **g**-tensor components larger than those of a tryptophan radical (Biskup et al., 2013). Published redox potentials and *g*-values lead to the conclusion that tyrosine is the only amino acid that is able to form such a RP (Bleifuss et al., 2001). A closer inspection of the *X*Cry structural model revealed two tyrosines, namely Tyr50 and Tyr397 to be ideal candidates to form a bridge between the FAD and the protein surface. In addition, these tyrosines are close to the Trp-triad and thus, are able to serve as a backup and function as an alternative electron transfer pathway. The assignment of the trEPR signals from the *X*Cry Trp377Phe and Trp400Phe mutants was again corroborated by spectral simulations, in which identical parameters as of the WT were used (Biskup et al., 2013). Only the **g**-tensor adjusted to be compatible with a tyrosine radical instead of a tryptophan radical. The resulting simulations showed signals shifted to lower magnetic fields, thus confirming a [Tyr···FAD] RP species. Another experimental way to prove this alternative electron transfer path was to investigate a Tyr50Phe/Trp400Phe double mutant, in which tyrosine and tryptophan were replaced by the redox-inert phenylalanine. Its weak trEPR signal was again shifted toward a higher magnetic field that closely resembles the WT signal, thus indicating a RP between Trp324 and the FAD.

**Figure 3:**
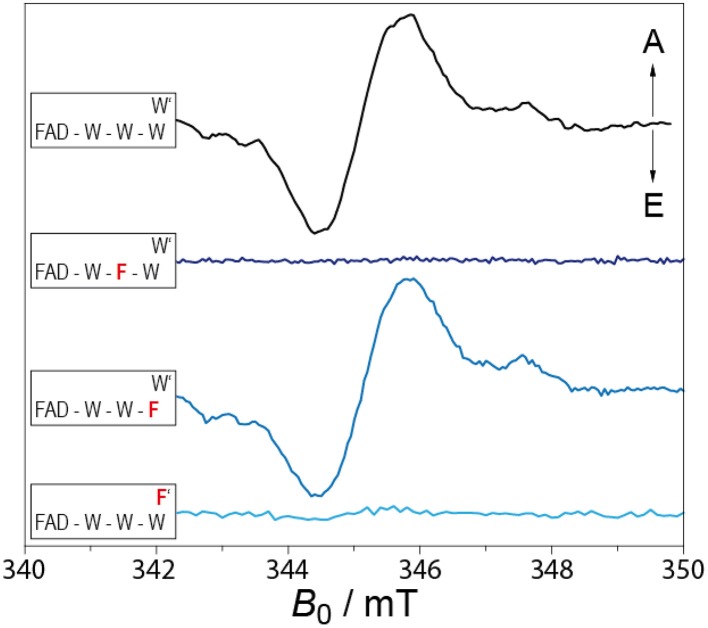
**TrEPR spectra of WT (solid black curve) and different Trp mutant proteins (solid blue curves) of *Synechocystis* Cry DASH**. The spectra were scaled to a comparable signal-to-noise ratio. The three tryptophans correspond to (Trp396, Trp373, and Trp320, W′ correspond to Trp375). From top to bottom: WT, Trp373Phe, Trp320Phe, and Trp375Phe. Adapted from Biskup et al. (2011).

In 2011, Biskup et al. expanded the concept of the highly conserved Trp-triad being the only electron-transfer pathway in the CRY/PL family even further (Biskup et al., 2011). Figure 3 depicts results from a trEPR study of wild-type and point-mutated CRY DASH protein from *Synechocystis* sp. (*S*Cry). Here, an alternative ET pathway was revealed because a point mutation of the terminal tryptophan (Trp320Phe) showed the same trEPR spectrum as compared to the WT protein. An investigation of the protein structure identified an alternative tryptophan (Trp375) that could function as terminal electron donor. However, with 8.2 Å between Trp375 and the middle tryptophan (Trp373), the alternative terminal Trp' is nearly 5 Å further apart from Trp373 as the “expected” conserved terminal Trp. To probe this suggestion a second point mutant, Trp375Phe, was produced. No trEPR could be recorded, even with the conserved Trp-triad being fully intact, thus indicating that in *S*Cry an alternative pathway for electron transfer is used. The spectral evidence is supported by calculations based on Marcus' theory of charge transfer (Krapf et al., 2012). Here the change in Gibb's free enthalpy for moving a positive charge along the conserved (Δ*G* = −62 kJ/mol) and alternative (Δ*G* = −81 kJ/mol) pathways were computed, showing that the stabilization energy is about 20 kJ/mol higher for the alternative Trp'. This demonstrates that distance is not necessarily the decisive parameter to determine the electron-transfer pathways in CRYs and PLs, but solvent accessibility, and the stabilization of the charge-separated states, as well as the relative orientation of the involved molecules contribute substantially.

With the new results from the last years, the picture of a RP being formed upon photoexcitation in CRYs seems to be widely proven. But different from the early expectations the conserved Trp-triad is not the only pathway for electrons from the protein surface to the FAD chromophore. Different paths and also different types of amino acids can be used for electron transfer, even with the classic Trp-triad being fully intact. Why different types of CRYs use different routes for the electrons is a question, which needs to be answered in the future.

### LOV domains

First discovered as tandem sensor domains in the plant photoreceptor phototropin (Christie et al., 1998), “Light-oxygen-voltage” (LOV) domains have since been found in several plant, fungal, and bacterial proteins (Christie, 2007). LOV domains constitute a subclass of the Per-ARNT-Sim (PAS) family whose members serve as versatile sensor and interaction domains in diverse signaling proteins (Möglich et al., 2009); their identified responses range from phototropism (Briggs, 2007) via entrainment of the circadian clock (Kim et al., 2007) to regulation of morphogenesis (Möglich et al., 2010). The first structure of a LOV domain was that of the LOV2 domain of *Adiantum capillus-veneris* phototropin (Crosson and Moffat, 2001, 2002), and it confirmed the canonical PAS fold. Residues involved in FMN coordination and the photoreaction are largely conserved and define the flavin-binding pocket (Krauss et al., 2009).

LOV domains are distinguished from other flavoproteins by their characteristic photochemistry. After absorption of a photon in the blue spectral region by the dark-adapted LOV-450 state, the FMN cofactor undergoes efficient intersystem crossing within nanoseconds to yield a LOV-700 intermediate state with FMN in its triplet state (Swartz et al., 2001; Kennis et al., 2003). Within microseconds a covalent thioether bond between atom C(4a) of the flavin ring and a conserved nearby cysteine residue is formed (a so-called cysteinyl-4a-adduct), the LOV-390 state (Salomon et al., 2000, 2001). The photoreaction is fully reversible, and the signaling state thermally reverts to the ground (dark) state.

In principle, adduct formation can occur via three different molecular mechanisms: a concerted mechanism, an ionic mechanism following initial protonation of the excited ^3^FMN triplet state, and a RP mechanism. To elucidate which leads to adduct formation, we designed a trEPR and optical study with various LOV domains at low-temperature: Triplet states of LOV domains were measured and compared to triplet states of flavins in solution (Kowalczyk et al., 2004; Schleicher et al., 2004). Specific protein-cofactor interactions that alter the electronic structure of LOV1 and LOV2 domains could be identified. In detail, a phenylalanine residue, which is highly conserved in LOV1 domains, alters the triplet-wavefunction (in particular the triplet “delocalization parameter” |*D*|) and is supposed to change the yield of adduct formation. Moreover, results from optical measurements clearly confirmed that adduct formation is possible at low temperatures, but the absorption spectra showed distinct changes as compared to spectra from frozen LOV domains with cysteinyl-4a-adduct. Additional triplet spectra were recorded at different pH values (in particular pH 2.8, which is well below the p*K*_a_ of 4.4 for the protonation of ^3^FMN, Schreiner et al., 1975), both with FMN in aqueous solution and bound to LOV domains. If a protonation of the FMN triplet would have occurred in the LOV domains prior to adduct formation at 77 K, then the resulting ^3^FMNH^+^ would have been visible in the trEPR spectra, and could easily be distinguished from ^3^FMN by its zero-field splitting parameters |*D*| and |*E*|, and also by its triplet relaxation time (Kowalczyk et al., 2004). TrEPR experiments on protein-bound FMN and in frozen aqueous solution confirmed that proton transfer to ^3^FMN does not occur at 77 K, which rules out the presence of ^3^FMNH^+^ as an intermediate at 77 K. These findings clearly demonstrated that adduct formation via an ionic mechanism can be excluded under these experimental conditions. Another possibility is that the thioadduct is formed via a concerted mechanism, directly from the triplet state. By the principle of conservation of angular momentum, however, the photoproduct is expected to be also formed in a triplet-spin configuration, and thus, should be detectable by trEPR; however, no such paramagnetic species was observed. This could be due to the fact that the triplet of the adduct species is hidden under the backdrop of an excess of triplets from unreacted LOV domains. It should be pointed out, however, that the triplet-state energy of the photoadduct is expected to be very high due to the formation of an sp^3^-hybridized C(4a) from a formerly delocalized, and hence, stabilized π-electron system of the isoalloxazine ring. Therefore, we consider a concerted mechanism via such a transition state rather unlikely.

The only remaining plausible mechanism is the RP mechanism: After electron transfer from the functional cysteine to ^3^FMN, a very short-lived and yet uncharacterized RP species is generated, which instantly forms a covalent bond after rapid triplet-to-singlet conversion. The generated protonated cysteinyl-4a-adduct deprotonates (an process inhibited at low temperature), and the signaling state is formed.

Despite closely similar sequence and almost identical structure, individual LOV domains differ markedly in their kinetics and quantum yields of the photocycles. Notably, dark-recovery times between a few seconds up to hours have been recorded (Kasahara et al., 2002; Zoltowski et al., 2009). One approach for understanding these phenomena is to assume that minor changes in the cofactor environment lead to subtle changes in protein conformation, which in turn could alter the stability of the carbon–sulfur bond, and thus, modulate the reaction speed of the ΔG-driven C–S bond splitting. A number of examples in the literature show significant modulation of adduct formation/bond breaking via point mutations at various sites of the phototropin protein (Christie et al., 2007; Jones et al., 2007; Raffelberg et al., 2011, 2013). Therefore, these findings support the idea that minute changes in LOV domains can lead to significant changes in their reactivity. For a profound understanding of this unique photochemical reaction, information on the hydrogen-bonding situation and the close environment of the photo-labile center is crucial.

For this purpose, a study has been designed where parts of the micro-environment in the close vicinity of the flavin cofactor of a LOV domain was altered and, via ENDOR spectroscopy, the influence on the protein's reactivity was elucidated (Brosi et al., 2010). Resolving the micro-environment of cofactors in proteins and its influence on biological function is difficult as most of these structural changes are far beyond the typical resolution of protein X-ray crystallography: To increase structural information extractable from experimental data, its temperature dependence needs also to be taken into account. From these data, the internal energy of the system, and hence, the strength of interaction between the cofactor and its surrounding can be estimated. For this purpose, site-directed mutagenesis of LOV domains where the reactive cysteine residue is exchanged with either alanine or serine, were devised. This prevents the formation of the C4a adduct and instead leads to a meta-stable FMNH^•^, which has been identified to serve as a reaction intermediate analog.

**Figure 4:**
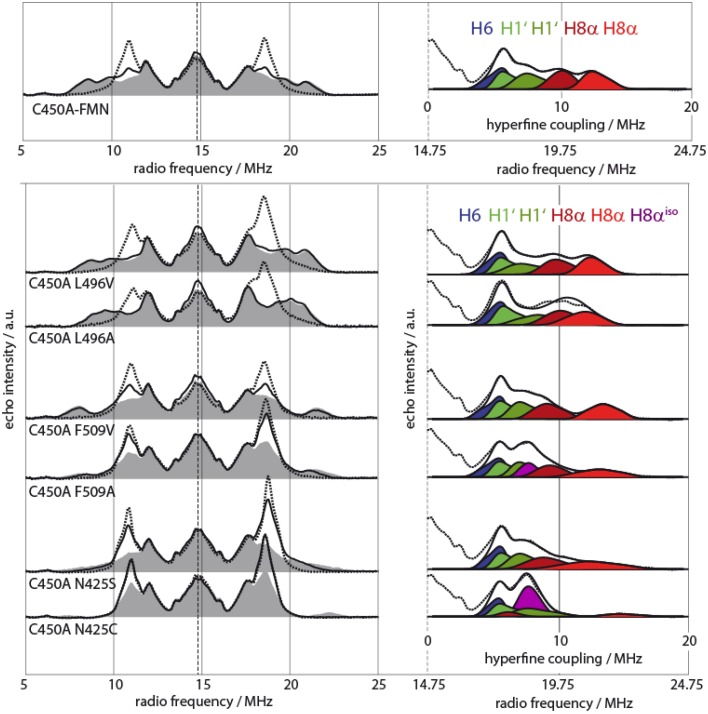
**Pulsed X-Band proton Davies-type ENDOR spectra of various *As*LOV2 single- and double mutants (adapted from Brosi et al., 2010)**. **Left**: Spectra were recorded at 120 K (dashed lines), 80 K (black lines), and 10 K (gray shaded) for all samples. **Right**: Sections of *As*LOV2 10-K spectra with accompanying spectral simulation of the outer wing of the spectrum (measured spectrum, dashed lines; single simulated hfcs, shades of blue, green, and red; envelope of simulated hfcs, black lines). Two protein samples, *As*LOV2 C450A/F509A and *As*LOV2 C450A/N425C, require another hyperfine component of axial symmetry for accurate spectral fitting. This feature represents hfcs from fast rotating 8α methyl group protons, is shown in purple and is denoted as H8α^iso^.

In detail, it was possible to detect a unique spectral behavior of *Avena sativa* LOV2 (*As*LOV2) C450A samples as their 8α methyl-group rotational motion is slowed down starting at already rather elevated temperatures (*T* < 110 K) (Brosi et al., 2010). To identify responsible amino acids for altered protein-cofactor interaction, an extended mutagenesis study has been performed with modifications introduced to in the direct vicinity of the 8α methyl-group, where three amino acids, namely Leu496, Phe509, and Asn425, are located (see Figure 4). Mutations in these three amino acids clearly showed changed temperature behavior, which is in line with the predicted altered sterical interaction (see Figure 4, left panel). Moreover, the hfcs from the three arrested 8α protons shift as depending on the individual mutants (see Figure 4, right panel). Spectral assignment of these hfc tensors in combination with DFT calculations resulted in the precise determination of the orientation of the methyl group with respect to the isoalloxazine ring plane.

Finally, as an important link between molecular spectroscopy and protein photochemistry, Asn425 was identified as a central amino acid for the photochemistry of LOV domains (Brosi et al., 2010). Kinetic measurements demonstrated that the *As*LOV2 Asn425Cys sample has a seven-fold shorter adduct-state life time as compared to *As*LOV2 WT. This is most likely due to altered Asn425–FMN interaction, which in the end destabilizes the intrinsically weak C–S bond of the cysteinyl-4a-adduct.

This type of study was recently repeated with an engineered LOV photoreceptor YF1 using optical and EPR spectroscopy (Diensthuber et al., 2014). To probe for benign or adverse effects on receptor activity, all amino acids surrounding the FMN were mutated and their impact on light regulation was determined. While several mutations severely impaired the dynamic range of the receptor (e.g., Ile39Val, Arg63Lys, and Asn94Ala), residue substitutions in a second group were benign with little effect on regulation (e.g., Val28Thr, Asn37Cys, and Leu82Ile). Both detection methods identified correlated effects for certain of the latter mutations on chromophore environment and response kinetics in YF1 and the LOV2 domain from *Avena sativa* phototropin 1.

In sum, both studies concluded that mutations close to the FMN cofactor provide a powerful tool to adjust the light-responses of LOV photoreceptors as demanded for optogenetic applications. As outcome from low-temperature ENDOR studies, a slight reorientation of the FMN binding position and as a consequence, a (de)stabilization of the C(4a) adduct seems to be the most likely effect.

### BLUF domains

In 2000, the most recent member of blue-light percepting proteins, the flavin-containing blue-light receptors exemplified in the N-terminus of the AppA protein of the purple bacterium *Rhodobacter sphaeroides*, was first described in the literature (Masuda and Bauer, 2002). AppA acts here as a transcriptional antirepressor and interacts with the photosynthesis repressor protein PpsR to form a stable AppA–(PpsR)_2_ complex in the dark. The blue-light activated form of AppA cannot associate with PpsR and thus, enables photosynthetic genes inhibition (Braatsch and Klug, 2004).

Via comparison of amino-acid sequences, a new class of blue-light photoreceptors, the so-called BLUF (“blue-light using FAD”) domains (Gomelsky et al., 2000), were introduced. This class contains a series of proteins from proteobacteria, cyanobacteria and some eukaryotes (Gomelsky and Klug, 2002). Three-dimensional structures from X-ray crystallographic data are now available for a number of BLUF proteins. Their arrangement is unique among flavin-binding proteins and bears a more global similarity to a ferredoxin fold rather than to other photoreceptor molecules (Anderson et al., 2005; Kita et al., 2005; Grinstead et al., 2006; Jung et al., 2006; Yuan et al., 2006).

Some sort of photocycle has been assumed for BLUF proteins, however, no specific structural changes upon illumination have been observed so far. Illumination of BLUF domains induces a small but distinct red shift of about 10 nm of the FAD absorption in the UV-visible region (Laan et al., 2003), which is reversible in the dark. Lifetimes between a few seconds and up to several hours have been determined (Kraft et al., 2003; Masuda et al., 2004). The photocycle is unique compared to the LOV photoreceptors, in which light absorption induces relatively large spectroscopic changes of the chromophore. Although the molecular mechanism of BLUF photochemistry is still under significant discussion (see, e.g., Domratcheva et al., 2008; Wu and Gardner, 2009), three conserved amino acids are believed to be essential for this light-driven reaction: a tyrosine, a tryptophan and a glutamine, which are all located in direct proximity to N(5) of the FAD's isoalloxazine moiety. A reaction scheme was first proposed by Gauden and coworkers (Gauden et al., 2005; Laan et al., 2006): following blue-light illumination, a transient RP comprising of the FAD and a conserved tyrosine is formed, which drives the hydrogen-bonding network around the N(5) position to rearrange. In contrast to the triplet intermediate state in LOV domains, the BLUF reaction starts out mainly from the excited singlet state of the FAD (the complete reaction cycle is proposed to be completed within 1 ns) (Gauden et al., 2005). Nevertheless, it has been demonstrated, that also triplet-state precursors are able to perform a hydrogen network rearrangement (Zirak et al., 2005).

As the primary light reaction is not accessible within the time scale of trEPR, light-activated intermediates of the FAD cofactor in three BLUF domains from *R. sphaeroides* (AppA-BLUF), *Synechocystis* sp. PCC 6803 (SlR-BLUF), and *Escherichia coli* (YcgF-BLUF) have been probed at low temperature (Weber et al., 2011). It was the goal of this study to shed some light on intermediate states, and to identify specific protein–cofactor interactions that modulate their electronic wave functions. As larger conformational changes are inhibited at these temperatures, the formation of intermediate states can be monitored; however, no final signaling state is formed thus rendering the reaction cyclic.

**Figure 5:**
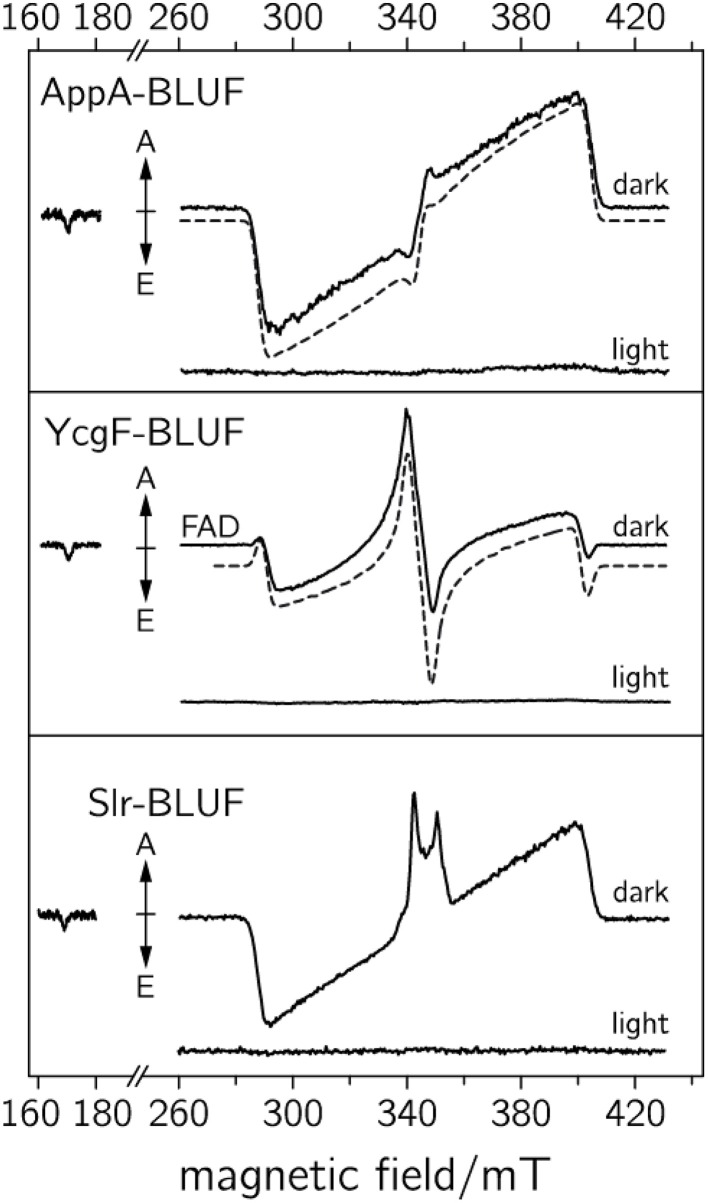
**TrEPR spectra of various BLUF domains recorded at 1 μs after pulsed laser excitation (adapted from Weber et al., 2011)**. **Upper**: dark-adapted and blue-light adapted AppA-BLUF protein. **Middle**: dark and blue-light illuminated YcgF-BLUF samples. **Lower**: dark and blue-light illuminated Slr-BLUF protein. The respective dashed curves represent spectral simulations of the dark-state sample.

Photo-induced flavin triplet states and RP species have been detected on a microsecond time scale, but revealed a completely different behavior as compared to previously investigated LOV domains (Schleicher et al., 2004). Moreover, BLUF domains exhibit much higher spectral diversity. While the trEPR spectrum of the AppA-BLUF protein (see Figure 5, upper panel) is very similar to that of the LOV domains, triplet-state spectra obtained from the YgcF-BLUF protein show a completely different electron-spin polarization pattern (see Figure 5, middle panel). Moreover, spectra recorded from the Slr-BLUF protein exhibit an even more complex spectral shape around *g* ~ 2 (see Figure 5, lower panel). To rationalize these differences, spectral simulations of flavin-triplet state trEPR spectra have been performed (see dashed lines in Figure 5) (Weber et al., 2011). It is interesting to note that pre-illuminated BLUF domains did not show any signal assuming an efficient deactivation process that is completed within a ns-time scale (Mathes et al., 2012).

TrEPR spectra from YcgF-BLUF domains led us to the assumption that a nearby methionine can alter the wave function of the flavin triplet state, and thus, can be responsible for the unusual electron-spin polarization pattern (Weber et al., 2011). Moreover, Slr-BLUF undergoes a competing electron-transfer reaction, which is tentatively assigned to a flavin-tyrosine RP, but deserves further investigation. Nevertheless, it remains unclear why only Slr-BLUF samples show light-induced electron transfer under the chosen experimental conditions. The answer to this question may be the key to correlating the electronic structure of light-induced excited states with biological signaling activity, which most likely depends on the stability of the light state. On the other hand, based on the rather broad trEPR RP signature, long-range electron transfer (over distances larger than 1 nm) can be excluded. Here, trEPR beautifully shows its potential for assigning electron-transfer partners even in molecules with several potential electron donors.

In a different approach, dark-state BLUF domains from *Thermosynechococcus elongatus* were illuminated at low temperature, and the resulting meta-stable radicals were characterized using steady-state EPR methods (Nagai et al., 2008; Kondo et al., 2011). The illumination at 5–200 K derived an EPR signal with a separation of 85 G between the main peaks around *g* ~ 2, showing a typical Pake powder pattern of magnetic dipole-dipole interaction between two nearby radicals. Extended illumination induced an EPR signal at *g* = 2.0045, which was assigned to a neutral flavosemiquinone FADH^•^. The Pake doublet was not detected in a mutant protein, in which the tyrosine residue was replaced with phenylalanine. Further analysis by pulsed-ENDOR spectroscopy revealed an assignment to an FADH^•^ and a tyrosine neutral radical by comparison with published ENDOR signals (Mino et al., 1997).

Very recently, a Gln50Ala mutant of Slr-BLUF was investigated (Fudim et al., 2015). Without the central glutamine, no red-shifted signaling state is formed, but light-induced proton-coupled electron transfer between the protein and flavin takes place, analogous as to the WT protein. Results from ultrafast optical spectroscopy demonstrated that the lifetime of the neutral flavin semiquinone–tyrosyl RP is greatly prolonged (from < 100 ps in the wild-type protein) to several nanoseconds, which indicates that the formation of radical intermediates drives the hydrogen-bond rearrangement in BLUF photoactivation. This lifetime is now in a range suitable for detection by trEPR; therefore, this method was applied to investigate this Gln50Ala mutation at ambient temperatures (Fudim et al., 2015). The resulting trEPR spectrum consists of a point-symmetric E/A/E/A signal pattern spanning a width of 15.5 mT, without any resolved hyperfine structure. From the symmetric pattern of the signal and its signature, a spin-correlated RP species with a singlet excited-state precursor can be assumed. This is in contrast to the aforementioned low-temperature spectrum of WT Slr-BLUF, which has been assigned to a triplet-based spin correlated RP. A comparison between the RP observed in BLUF domains and cryptochromes, respectively, reveals major differences regarding signal pattern and width. For a quantitative analysis of the spectra obtained from the Slr-BLUF Gln50Ala sample, however, spectral simulations of this, for the first time in a protein observed strongly-coupled RP have to be performed to obtain exact distances and radical compositions.

### Summary

**Figure 6:**
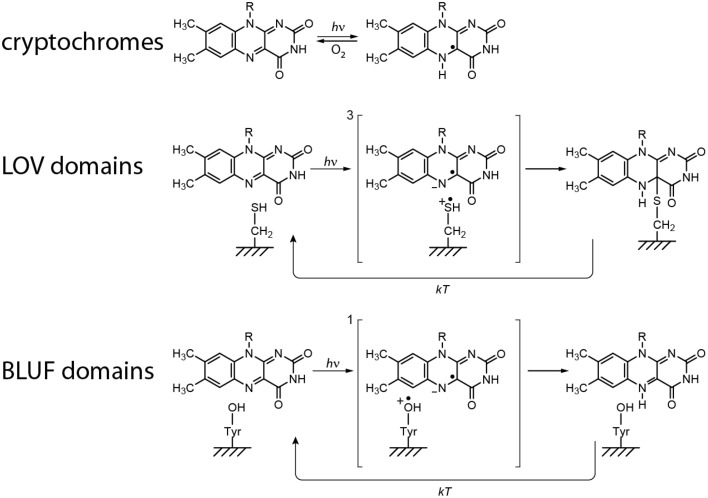
**Proposed primary light reactions of flavin-dependent photoreceptors**.

The to date proposed and commonly accepted primary light reactions of the three examined flavin-dependent photoreceptors are summarized in Figure 6. Both differences as well as commonalities between each of the molecular mechanisms can be detected: Whereas all signaling states are in principle reversible, only the CRY reaction comprises of a redox reaction and thus, a yet unidentified electron donor is required in order to facilitate signaling-state formation *in vivo* (established electron donors like EDTA or DTT are used for *in vitro* measurements). This in turn demands an oxidizing agent (most likely molecular oxygen in case of *in vitro* measurements) for back reaction to the dark state. In contrast, no additional reagents could yet be identified for back reaction in BLUF and LOV domains. Their signaling states are intrinsically metastable and thus, back reactions are only dependent on the thermal energies of the system. Consequently, the back reaction of CRYs directly depends on the accessibility and on the concentration of the oxidizing agent, whereas the life times of the signaling states in LOV and BLUF domains can be significantly modulated by the amino acids surrounding the flavin cofactor.

On the other hand, all mechanisms unify an electron transfer reaction after light excitation, very likely because of the large oxidation potential of excited-state flavins, which is estimated to be around 1.8 V (Heelis et al., 1990). However, clear differences of the life times and the distances between the three intermediate RPs are obtained. Whereas, CRY RPs are ~20 Å apart, and hence, have millisecond life times, distances of ~6 Å and correspondingly short life times are obtained in BLUF and LOV domains.

In sum, investigations with EPR spectroscopy are an obvious choice and indeed, played a substantial role for the elucidation of the primary reactions of these photoreceptors. In detail, first mechanistic proposals based on results from EPR (and optical) investigations were published for CRYs and LOV domains (Schleicher et al., 2004; Banerjee et al., 2007; Bouly et al., 2007). In addition, trEPR has been proven valuable for characterization of electron-transfer channels and the participating amino acids (Biskup et al., 2009, 2011, 2013; Weber et al., 2010). The signaling-state generation in BLUF domains, however, is generated within only a few nanoseconds, which inhibit direct results from EPR spectroscopy. In this case, either slowing down the reaction by decreasing the temperature or altering the reaction by selective mutations has been used.

## How can secondary events like conformational changes be investigated by molecular spectroscopy?

The signal-transduction events following the primary photo-processes are far from being fully understood in all three photoreceptor classes, although some key results emerge to shed light onto the principal mechanisms: More than 10 years ago, ground-breaking results from NMR spectroscopy demonstrated a reordering or even an unfolding of the helix (named *J*_α_) bridging a LOV2 domain and its signal-transduction module (Harper et al., 2004a,b; Herman et al., 2013), and thus, displayed a first molecular concept for signal propagation. Subsequent experiments with other LOV domains yielded evidence that also dimerization reactions can occur after light excitation (Buttani et al., 2007; Möglich and Moffat, 2007; Nakasako et al., 2008; Zayner et al., 2012; Herman et al., 2013). In *Arabidopsis* CRY2, a homo oligomerization and subsequent binding to its partner protein CIB1 has been identified (Hulsebosch et al., 1997; Kennedy et al., 2010), however, the variability of the CTT domain in CRYs implies that also other mechanisms are possible. In BLUF domains, also monomerization reactions have been identified (Prisner et al., 2001; Yuan et al., 2006; Wu and Gardner, 2009).

Despite these initial achievements in unraveling signal-transduction pathways, several major questions regarding these secondary steps in photoreception remain unanswered: Up to now, the interaction between individual multi-domain photoreceptors (e.g., LOV1 and LOV2) and how the two domains modulate the light-sensing signal are still unknown. Moreover, the slow dark-state recovery and the remarkable divergence of this reaction depending on the organism are still far from being understood. Furthermore, most amino acids that are responsible for conformational changes remain unidentified. Knowledge of these is in turn essential for application to optogenetics: exact control of the photoactivation and photoreversion rates, and optimized affinities between photoreceptor and signaling domain are highly desired and necessary.

In principle, photoreceptors are ideal for examining conformational changes as (i) both dark and signaling states can be generated with almost 100% yield; subsequent temperature reduction enables freezing the light state and allows for steady-state spectroscopy on both states, (ii) all reactions are in principle reversible, which permits accumulation of signals from intermediate states, and (iii), short light pulses can be perfectly used as intrinsic trigger for time-resolved spectroscopy. The application of structure determination methods such as X-ray crystallography or NMR spectroscopy, however, are limited for the analysis of the signaling states because (i) dimerization reactions and/or reordering of domains “precludes” crystallization and (ii), most photoreceptors are too large for NMR spectroscopy. On the other hand, EPR spectroscopy in combination with SDSL may help to uncover conformational changes in photoreceptors. To examine structural changes upon light excitation, various EPR experiments are conceivable.

In general, the room temperature EPR-spectral shape of a nitroxide SL is sensitive to the reorientational motion of its side chain due to partial motional averaging of anisotropic components of the **g**− and hfc-tensors (Klare and Steinhoff, 2009; Hubbell et al., 2013). Therefore, changes in mobility, accessibility and polarity can be used to characterize the effects on the EPR signature due to the motional rate, amplitude, and anisotropy of the overall reorientational motion (Marsh, 2010; Klare, 2013). Changes in these parameters result in altered *g*-factors, spectral line widths, hf splittings, relaxation times, and rotational correlation times; all of them can be extracted from an EPR spectrum via spectral simulations (Klare and Steinhoff, 2009; Klare, 2013). This concept can be applied to all photoreceptors as changes in mobility of dark− and signaling state can be probed via cwEPR at ambient temperatures. To do so, a number of SDSL proteins have to be produced, and differences between the two states mapped. With the help of modern computational chemistry, changes of EPR parameters can nowadays be correlated to structural changes (Mchaourab et al., 2011; Polyhach et al., 2011). The drawback of this method is that only qualitative conclusions are possible.

For a quantitative analysis of conformational changes, the aforementioned concept has to be expanded to doubly-SL proteins. The basis for this approach is that the distance between the two SLs can be determined precisely through quantification of their mutual spin–spin interaction. Spin–spin interaction comprises static dipolar interaction, modulation of the dipolar interaction by the residual motion of the spin label side chains, and exchange interaction. The combination of static dipolar and exchange interaction in an unordered, immobilized sample leads to considerable broadening of the cwEPR spectrum if the interspin distance is less than 2 nm. In this case, interspin distances can then be determined by a detailed line-shape analysis of EPR spectra of frozen protein samples using spectra- deconvolution; however, this approach is still prone to various sources of errors (Hubbell and Altenbach, 1994; Hubbell et al., 2000; Steinhoff, 2004).

**Figure 7:**
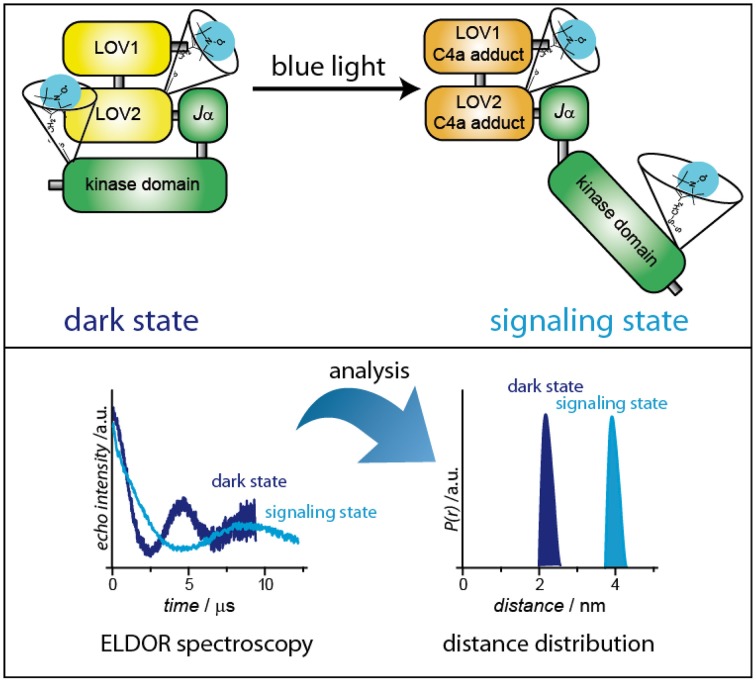
**Distance determination of dark- and signaling-states via pulsed ELDOR spectroscopy**. As a representative example, plant phototropin has been chosen.

On the other hand, exchange interaction is typically negligible for interspin distances larger than 2 nm. Here, the direct measurement of the dipolar coupling is possible with the DEER/PELDOR pulse sequence, which separates the dipole–dipole interaction from other contributions to the spin Hamiltonian. DEER/PELDOR is based on the separate excitation of two groups of electron spins by applying 2 mw frequencies. The strength of the dipolar interaction is directly observed as modulated decay curves in the time domain (Figure 7). Via Fourier transformation, the frequency of the modulation can be extracted and directly correlated to the interspin distance (Polyhach et al., 2011; Jeschke, 2012). Comparison of distances between dark- and signaling states, performed with a number of differently doubly SL photoreceptors, results in a mapping of distance distributions that correspond to the extent of conformational changes. As a representative example, the expected alteration of the distance distribution in phototropins is depicted in Figure 7. The aforementioned reordering of the *J*_α_-helix is expected to result in a displacement of the kinase domain. If SLs are attached to one of the LOV domains and to the kinase domain, these structural changes can be monitored and analyzed. Although the accessibility of full-length structural information is very helpful for the analysis of distance data, interpretation is still possible without this information at hand: A combination of structural information of the individual domains and modern structure modeling is able to bypass missing full-length structures. It has to be mentioned that this concept can be easily adapted to follow dimerization (monomerization) reactions: Here, the photoreceptor is labeled at one position, and DEER/ELDOR spectra of dark- and signaling states are recorded. Distances will only be obtained when the photoreceptor is in its dimeric state.

The only disadvantage of the concept is that for efficient and selective coupling of the SL to the protein, cysteine residues at defined positions are obviously prerequisite. In case of small LOV and BLUF domains, no or only a few cysteines are encoded in the WT proteins; however, a number of naturally occurring cysteine residues are found in large photoreceptors such as phototropins or plant cryptochromes. Here, “unwanted” cysteines need to be removed with the help of molecular-biology methods prior labeling to avoid attachment of more than two SL thereby complicating distance analysis. Although the general procedure is well established, mutating a number of amino acids in a protein may always lead to misfolded and/or non-functional protein. But such effects can in principle be controlled by a “clever” mutation scheme.

Additionally, various recently developed labeling techniques using click-chemistry and unnatural amino acids enable the possibilities to circumvent general labeling problems such as cysteine-rich proteins (Fleissner et al., 2009). Moreover, the usage of orthogonal labeling with two different SLs attached to the protein of interest provides additional information and clear advantages in performing ELDOR/DEER spectroscopy as modulation depths of the recorded time traces are significantly increased and selective mw excitation can be applied (Chen et al., 2011).

Clearly, bringing together modern pulsed EPR, ideally performed at various magnetic-field/microwave-frequency ranges to exploit orientation-selection effects, with other nowadays well established methods such as SDSL, freeze quenching, molecular quantum mechanics, molecular mechanics, and, last but not least, sophisticated frequency analysis to accurately extract SL distances, provides the means of unraveling the structure and structural changes of photoreceptors, isolated and in complex with other proteins. Although this global combined approach is challenging and not straightforward to apply, it may be the only means in cases where other more highly resolving techniques, such as X-ray diffraction, due to the lack of high-quality single crystals, or NMR, due to the too large size of a protein complex, do not lead to meaningful propositions for a full comprehension of the functioning of photoreceptors. While EPR and in particular trEPR have already demonstrated their value in unraveling the primary photochemistry of blue-light photoreceptors, DEER/PELDOR still needs to prove itself in the vibrant but challenging photoreceptor research field. Nevertheless, we are confident, that within the next decade significant insight in signal transduction will be presented based on pulse EPR distance analyses.

## Funding

Deutsche Forschungsgemeinschaft, Research Training Group RTG 1976 (project 13).

### Conflict of interest statement

The authors declare that the research was conducted in the absence of any commercial or financial relationships that could be construed as a potential conflict of interest.
